# Supply chain management and accessibility to point-of-care testing in resource-limited settings: a systematic scoping review

**DOI:** 10.1186/s12913-019-4351-3

**Published:** 2019-07-24

**Authors:** Desmond Kuupiel, Vitalis Bawontuo, Paul K. Drain, Nonjabulo Gwala, Tivani P. Mashamba-Thompson

**Affiliations:** 1 Department of Public Health Medicine, School of Nursing and Public Health, University of KwaZulu-Natal, Durban, South Africa; 2 Faculty of Health and Allied Sciences, Catholic University College of Ghana, Fiapre, Sunyani, Ghana; 3 International Clinical Research Center, Department of Global Health, University of Washington, Seattle, USA; 4 Division of Infectious Diseases, Department of Medicine, University of Washington, Seattle, USA; 5 Department of Epidemiology, University of Washington, Seattle, USA

**Keywords:** Point of care diagnostics; supply chain management; accessibility, Availability, Use, Low-and-middle-income countries

## Abstract

**Background:**

World Health Organization (WHO) has created an essential list of in-vitro diagnostics. Supply chain management (SCM) is said to be the vehicle that ensures that developed point-of-care (POC) tests reach their targeted settings for use. We therefore, mapped evidence on SCM of and accessibility to POC testing (availability and use of POC tests) in low- and middle-income countries (LMICs).

**Methods:**

We conducted a systematic scoping review using Arksey and O’Malley’s framework as a guide. We searched PubMed; CINAHL; MEDLINE; WEB of Science; Science Direct; and Google Scholar databases for studies that focused on POC diagnostic tests and SCM. The review included studies that were undertaken in 140 countries defined by the World Bank as LMICs published up to August 2017. Two reviewers independently screened the abstracts and full articles against the eligibility criteria. The study used the mixed methods appraisal tool version 2011 to assess the risk of bias for the included studies. NVivo version 11 was employed to extract themes from all included studies and results presented using a narrative approach.

**Results:**

Of 292 studies identified in this review, only 15 published between 2009 and 2017 included evidence on POC diagnostics and SCM. Of the 15 studies, three were conducted in Zambia, one each in Mozambique, Uganda, Guatemala; South Africa, one in Burkina Faso, Zimbabwe, and one multi-country study (Tanzania, Uganda, China, Peru and Zambia and Brazil). Six studies were not country specific since they were not primary studies. Majority of the studies reported stock-outs of HIV, syphilis, and malaria POC tests. There was a moderate to substantial level of agreement between the reviewers’ responses at full article screening stage (Kappa statistic = 0.80, *p* < 0.01). Nine studies underwent methodological quality appraisal and all, scored between 90 and 100%.

**Conclusions:**

The results demonstrated limited published research on SCM of and accessibility to POC testing in LMICs. Further studies aimed at investigating SCM of POC tests in resource-limited settings to identify the barriers/challenges and provide a context-specific evidence-based solutions for policy/decision makers, implementers, and POC developers, funders, and development partners would be essential.

**PROSPERO registration number:**

CRD42016043711.

**Electronic supplementary material:**

The online version of this article (10.1186/s12913-019-4351-3) contains supplementary material, which is available to authorized users.

## Background

Access to accurate, safe and appropriate point-of-care (POC) testing is vital for routine care, early diseases, and monitoring of diseases [1]. A POC diagnostic tests refer to medical device used for detection, diagnosis, and monitoring of diseases at the POC or near where healthcare service is provided [2–6]. POC testing deliver prompt results for clinical decisions as well as early referrals [4]. Access to POC testing services reduces test result turnaround times and facilitates prompt disease diagnosis and treatment initiation [6]. World Health Organization (WHO) encourages countries to establish their own essential diagnostic list based on the country’s disease burden and epidemiology to improve access to healthcare services [7]. Access to POC testing services can improve health outcomes particularly, in resource-limited settings.

Accessibility to POC diagnostic services is dependent on many factors, including an efficiently managed SCM, which is agile, flexible and responsive [8, 9]. Efficiently managed SCM ensures that appropriate POC diagnostics are available for use by health care professionals to perform test for patients who need it [1, 10]. This study defined SCM according to Pinna et.al. (2015) and Management Science for Health (2012) [10, 11]. SCM of POC diagnostic tests is therefore, defined to include POC test development, selection, forecasting and quantification, procurement, quality assurance, distribution, inventory management, redistribution, usage, and safe disposal of used test kits at the POC [10, 11]. A poor supply chain system may pose a threat to the availability and POC tests usage in rural primary health care facilities [9, 12, 13]. Poor SCM of POC diagnostics could also affect the implementation and sustainability of POC diagnostic services for vulnerable populations such as pregnant women in hard-to-reach communities [14–16] and may result in poor accessibility to POC diagnostic services for essential care [17–19]. Thus, health care SCM is more complex when compared to other industries because of its impact on people’s health [20].

Considering the potential challenges SCM could pose to accessibility of POC diagnostic services particularly in rural communities in LMICs, POC test availability and use, there is an urgent need to explore the role of SCM to help address supply chain barriers of POC diagnostic services in resource-limited settings and rural primary health care clinics. Therefore, this study aimed to map evidence on SCM of and accessibility to POC testing focusing on availability and use of POC tests in LMICs.

## Methods

### Purpose of the scoping study

We explored SCM and accessibility to POC testing using a scoping review methodology and adduced evidence on availability and use of POC tests in LMICs. A scoping study is useful and enables the identification of research gaps by mapping literature on a research question of interest as prescribed by the enhanced 2005 Arksey and O’Malley’s framework [21, 23]. A scoping review study prior to the conceptualization of a primary research question or a systematic review and meta-analysis may also be useful. We adopted the Preferred Reporting Items for Systematic Reviews and Meta-Analyses [22], and the enhanced Arksey and O’Malley’s framework [21, 23] to guide this study. Prior to the conduct of this study, we registered the review protocol in international prospective register of systematic reviews (CRD42016043711) [24].

### Identifying the research question

The main review question was: What is the evidence on supply chain management and accessibility to POC testing services (availability and use) in LMICs?

The sub review questions were as follows:

What is the evidence on availability of POC tests in LMICs?

What is the evidence on use of POC tests in LMICs?

Population, Intervention, Comparison, and Outcome (PICO) framework was used to determine the eligibility of the primary research question as shown in Table 1.

**Table 1:** PICO framework for defining the eligibility of the studies for the primary research question

P-Population	All types of point of care diagnostics
I-Interventions	Supply chain management (production, selection, quantification, procurement, storage, distribution, redistribution, quality assurance, inventory management, negotiation with suppliers, and safe disposal of use POC diagnostics)
C-Comparison	Absent supply chain management measures
O-Outcomes	Availability of POC diagnostic tests Use of POC diagnostic tests

### Literature search

We conducted a systematic literature search from the following databases: PubMed; EBSCOhost (CINAHL and MEDLINE); WEB of Science; Science Direct; and Google scholar. The database search occurred in June up to August 2017 using the following keywords: “diagnostics” “supply chain” “POC diagnostics” “supply chain management” (Additional file 1). Boolean terms (AND/ OR) were used to separate the keywords. Date, language, and study design restrictions were removed to broaden the search and capture the full range of literature on POC diagnostics and supply chain management.

### Study selection

Guided by the eligibility criteria, DK conducted the database search and screened the titles. DK and NG then independently screened the abstracts and full articles in parallel. At the abstract screening stage, differences in reviewers’ responses were discussed by the review team until consensus was reached. However, a third reviewer (BV) addressed all discrepancies at the full article screening stage.

### Eligibility criteria

#### Inclusion criteria

Included studies that met the following criteria:

- Evidence of POC diagnostics
- Evidence of the study been conducted in a LMIC
- Evidence of POC diagnostics production
- Evidence of POC diagnostics selection
- Evidence of POC diagnostics quantification
- Evidence of POC diagnostics procurement
- Evidence of POC diagnostics storage
- Evidence of POC diagnostics distribution
- Evidence of POC diagnostics redistribution
- Evidence of POC diagnostics quality assurance
- Evidence of POC diagnostics inventory management
- Evidence of negotiation with suppliers for POC diagnostics supply
- Evidence of safe disposal of used POC diagnostics products
- Evidence of POC diagnostics accessibility
- Evidence of POC diagnostics availability
- Evidence POC diagnostics use

#### Exclusion criterias

A study was excluded if it did not meet the inclusion criteria. The exclusion criteria included the following:

- Lack of evidence of POC diagnostics
- Studies conducted in high income countries
- Laboratory based POC diagnostics
- Studies that do not report on the primary outcomes of the study

### Charting the data

The selected studies were thoroughly read for data extraction of bibliographic details, aim/objective, study design, targeted population, intervention(s), relevant outcome(s), study setting, type(s) of POC diagnostics used, SCM interventions, and relevant outcomes of interest were extracted. Other information such as geographical location (rural or urban), country of the study, as well as, funding sources were also extracted. Data on country levels of income were obtained from The World Bank Group July, 2017 open data [25].

### Quality of the evidence

We used the 2011 version of the mixed methods appraisal tool (MMAT) [26] to appraised all included primary studies. We appraised the included primary studies under the appropriate study designs as prescribed by the MMAT. DK and BV appraised the studies and areas of disagreement in ratings were resolved through discussion. A quality percentage score of ≤50%, 51–75%, 76–100%, was interpreted as low quality, average quality, and high-quality respectively.

## Results

**Fig. 1:**
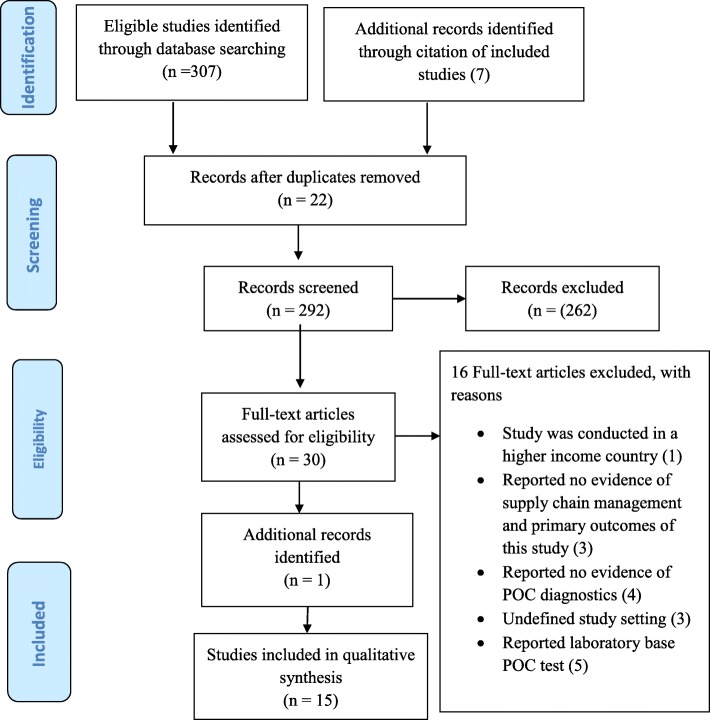
PRISMA 2009 Flow Diagram

Two hundred and ninety-two (292) articles met the eligibility criteria following deletion of 22 duplicates from the total 314 articles identified at the title screening stage (Fig. 1). Subsequently, 262 and 16 articles were also excluded following abstract and full screening respectively. Reasons for the exclusion after full article screening were: one study was not conducted in a LMIC [27], two studies reported no evidence of both supply chain management or the primary outcomes of this study [28–30]; four studies reported no information for POC diagnostics [31–34]; three studies had undefined study setting [35–37] and; five studies reported laboratory-based POC testing [38–42]. In all, 15 studies met the study eligibility criteria for data extraction including one study identified following an updated searched. There was a moderate to substantial level of agreement between the reviewers’ responses at full article screening stage (Kappa statistic = 0.80, *p* < 0.01) (Additional file 2). Thematic content analysis was conducted using the following themes: POC diagnostics accessibility; availability and; usage. NVivo version 11 was employed to extract themes from all included studies and results presented using a narrative approach. Emerging themes were also reported.

### Characteristics of included studies

**Fig. 2:**
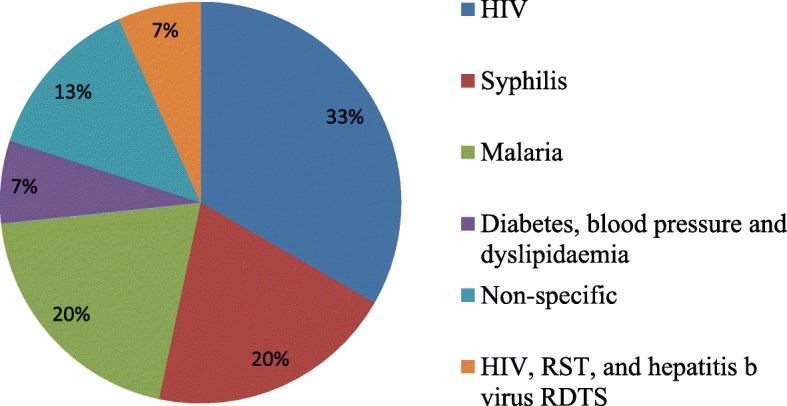
Population of POC test

**Fig. 3:**
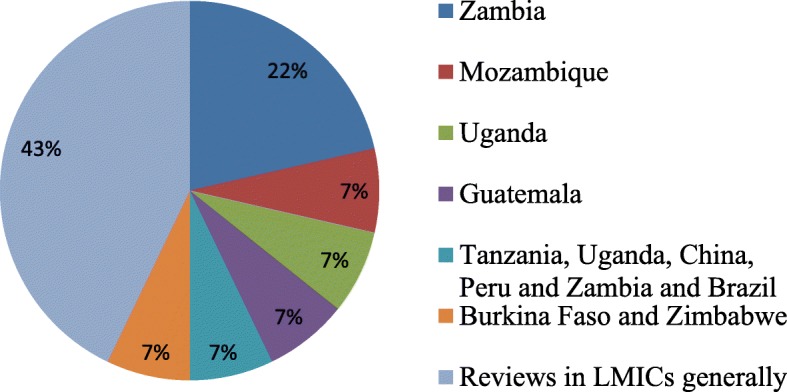
Distribution of countries where included studies were conducted

Tables 2 and 3 summarises the characteristics of the 15 included studies and findings. The 15 studies comprised of: one cluster randomized controlled trial [43], three cross-sectional studies [44–46], one quasi-experimental study [47], two mixed methods studies [48, 49], one cohort study [50], one qualitative study [51], and six expert reviews [52–57]. All included studies were published in English language between 2009 and 2017. All included studies reported on different POC diagnostic algorithms. Of the 15 studies, five studies were on HIV POC testing [46, 50, 52, 53, 56], three on rapid syphilis testing (RSTs) [47, 49, 51], three on malaria RDTs [43, 45, 48], two reported generally on POC diagnostics [54, 57], one on diabetes, blood pressure and dyslipidaemia assays [55], and one on HIV, RST, and hepatitis b virus RDTS [44]. Seven studies were conducted in rural settings [43–46, 50, 51, 56], and eight studies in both rural and urban [47–49, 52–55] (Fig. 2). Of the 15 studies, three were conducted in Zambia [43, 47, 51]; one in Mozambique [48], one in Uganda [45], one in Guatemala [44], one in Tanzania, Uganda, China, Peru and Zambia and Brazil [49], one in South Africa [46], and one in Burkina Faso and Zimbabwe [50], and six studies were not country specific since they were not primary studies [52, 54–57] (Fig. 3).

**Table 2:** Characteristics and finding of studies included in this scoping review

Author and year	Target Population	Type of POC diagnostic	Supply chain management measures reported	Desirable outcome
Alemnji et al., 2011 [56]	General population	HIV	Challenges in procurement, reagent inventory and stock maintenance; timely and quality testing; and challenges with who manages the supply chain systems	Affected confidence in test results and patient care; huge challenges with accessing testing services when services are available at the national level.
Ansbro et al., 2015 [51]	Pregnant women	Syphilis RSTs	Quality assurance activities and supervision	Reduced clinic waiting time, travel time and increased case detection and treatment, Acceptability and usability of RST kits and quality assurance activities, supply of RST kits less reliable, and stock-out
Bonawitz et al., 2015 [47]	Pregnant women	Syphilis RSTs	None reported	High levels to complete stock-outs at baseline, midline and end line periods over several weeks
Hamer et al., 2012 [43]	children < 5 years	Malaria RDTs	Transparent record keeping, adequate supplies, stock management, daily registers and periodic reconciliation of stocks, and ensuring that none had passed their expiration dates	Availability and use of malaria RDTs with over 98% accountability of the RDTs
Hasselback et al., 2014 [48]	General population	Malaria RDTs	Analysis of distribution system characteristics	High levels of stock-out
Kyabayinze et al., 2012 [45]	General population	Malaria Microscopy and RDTs	None reported	limited availability of RDTs, limited use of RDTs, lack storage space, and lack of glucometers
Mabey et al., 2012 [49]	Pregnant women and Sexually active populations	Syphilis RDTs	Training in stock management, record keeping, and quality control, monitoring supply chain problems and provision of sustainable solutions in case of stock-outs	improve access, increase antenatal clinic attendance, availability and use of RSTs
McGuire et al., 2014 [55]	General population	Diabetes, Blood Pressure, and Dyslipidemia assays	Reported frequent challenges with distribution of devices	Stock-outs and limited use of devices
Peeling, 2015	General population	Not specified	Called for redistribution when necessary	Prevent diagnostics from expiring
Peeling and Ronald, 2009 [54]	General population	Not specified	Reported supply chain failure	Test stock-outs
Shott et al., 2012 [53]	General population	HIV	Challenges with quality management (QM)systems	Good QM ensures accuracy of devices; transform the availability of tests in real time; and inform proper patient care.
Smith et al., 2015 [44]	Pregnant women	HIV, Syphilis, and HBV RDTs	No supply chain management measures	Increased coverage increase uptake, test stock-outs, limited uptake, and low testing
Stevens et al., 2014 [52]	General population	HIV viral load	Raises challenges with reimbursement, quality monitoring, lack guideline and regulations	Where VL testing is available, frequency of CD4 monitoring is reduces or stopped altogether
Thairu et al., 2011 [50]	General population	HIV (CD4 testing with Guava EasyCD4	Guava offered to provide a robust supply chain for reagents and maintenance	Increase access to CD4 testing, low repeated testing, poor stock management, tardy response from Guava resulting in lost operating time
Jaya et al., 2017 [46]	Clinics	HIV rapid testing kits	Compliance to guidelines for purchasing and inventory.	HIV rapid test kits shortage in 4 clinics

**Table 3:** Characteristics of included studies and findings

Author and date	Country	Geographical area	Study setting	Study design
Alemnji et al., 2011 [56]	Developing countries	Rural	Resource limited settings	Expert review
Ansbro et al., 2015 [51]	Zambia	Rural	Antenatal clinic	Mix method
Bonawitz et al., 2015 [47]	Zambia	Urban and Rural	Antenatal clinic	Quasi-experimental evaluation
Hamer et al., 2012 [43]	Zambia	Rural	Children clinic	Cluster-randomised controlled trial
Hasselback et al., 2014 [48]	Mozambique	Urban and Rural	General clinics	Mix method
Kyabayinze et al., 2012 [45]	Uganda	Rural	General clinic	Cross-sectional survey
Mabey et al., 2012 [49]	Tanzania, Uganda, China, Peru and Zambia and Brazil	Urban and Rural	Antenatal clinics and Community-based	Mix Method
McGuire et al., 2014 [55]	Developing Countries	Urban and Rural	Primary and secondary health facilities	Narrative review
Peeling, 2015	Developing Countries	Urban and Rural	Resource limited settings	Expert review
Peeling and Ronald, 2009 [54]	Developing Countries	Urban and Rural	Resource limited settings	Narrative Review
Shott et al., 2012 [53]	Sub-Sahara Africa	Urban and rural	Resource limited settings	Expert review
Smith et al., 2015 [53]	Guatemala	Rural	Antenatal clinics and Community-based	Cross-sectional survey
Stevens et al., 2014 [52]	Developing countries	Urban and rural	Resource limited settings	Expert review
Thairu et al., 2011 [50]	Burkina Faso and Zimbabwe	Rural	Hospital and community-based clinic	Cohort study
Jaya et al., 2017 [46]	South Africa	Rural	Primary Healthcare Clinics	Cross-sectional (Audit)

### Quality of evidence

Of the 15 studies, nine underwent methodological quality assessment [43–45, 47–51] using the MMAT – Version 2011 [26]. The remaining six were excluded because they were not primary studies [52–57]. The nine studies which underwent methodological quality appraisal scored between 90 and 100%. Of these, six studies scored the highest quality score of 100% [43–48]. Two studies scored an average of 91.7% [50, 51], and the remaining one study scored 90% [49].

### Study findings

#### Availability of POC diagnostics

A total of 13 studies reported on availability of POC diagnostics in LMICs [43–48, 50, 52–56]. Of the 13 studies, 11 reported stock-out of various POC diagnostics including: RSTs, malaria RDTs, HIV, hepatitis b virus, blood pressure, diabetes, and dyslipidaemia POC assays [43–48, 50, 51, 53–55].

##### Availability of rapid syphilis tests

A study aimed at comparing healthcare workers experiences and challenges in scaling up from a highly supported non-Government Organization-led pilot to a large-scale ministry of health-led national programme in Zambia has shown that half of the piloted sites reported a stock-out on one occasion [51]. It was also shown that almost a third of rollout sites reported a complete stock-out of RSTs during the month preceding interviews [51]. Another study in Zambia that evaluated the impact of RST and treatment in pregnant women also reported stock-out of RSTs at various stages [47]. A total of 33.3% (2/6) of the facilities documented stock-outs during the baseline ranging from 8 to 20 weeks, 33% (5/15) during the midline period ranging from 4 to 12 weeks with a median stock-out period of 6 weeks; and 60% (9/15) during the end line period ranging from 1 to 16 weeks, with a median stock-out period of 5.5 weeks [47]. Peeling and Ronald (2009) further reported that RSTs stock-out was one of the causes of poor levels of syphilis testing among pregnant women in LMICs [54]. The findings showed weaknesses in the supply chain system such as procurement, inventory and stock management, and human resource capacity for SCM during implementation hence, resulted in significant RST stock-outs.

##### Availability of malaria RDTs

A substantial high levels of malaria RDTs stock-out was reported in Capo Delgado province in Mozambique [48]. This high levels of stock out was associated with poor test usage [48]. Estimated loss of consumption percentage for malaria RDTs were significantly high with a weighted average of 78% [48]. Increased rates of stock-outs were observed with increasing levels of consumption [48]. In a study aimed at assessing health system’s capacity to absorb Parasite based Malaria Diagnosis in Uganda has shown that, malaria RDTs were available only in 24% (30) out of the 125 lower level health facilities [45]. The study also reported limited availability of malaria RDTs, as well as lack of glucometers [45]. A survey aimed at assessing the quality and safety of having community health workers (CHWs) use RDTs and provide integrated management of malaria and pneumonia revealed 72.2% of CHWs did not receive malaria RDTs kits for 6 months [43].These findings demonstrate limited availability of malaria RDTs, stock out of malaria RDTs, and lack of glucometers in the study settings. The findings also demonstrate that further research is needed to determine the impact of malaria RDTs stock outs on patients’ outcomes in these settings.

##### Availability of HIV test, HBV test, and other assays

A study conducted in rural antenatal clinic and community- based settings in Guatemala showed that nearly half of the women who turned up for HIV, syphilis, and hepatitis b virus testing services did not get tested partly due to stock-outs [44]. Another study has also demonstrated the prevalence of blood pressure monitoring devices, diabetes, and dyslipidaemia POC assays stock-outs in LMICs [55]. It was reported that manufacturers of new assays entering the market are most often unable to meet the demand of rapid recommendations [52]. A delayed response by Guava trained technicians resulted in Guava EasyCD4 downtimes of 8 out of 45 months, nearly 18% of the total available operating time was reported [50]. In addition, poor stock management resulted in expiration of Guava EasyCD4 since the shelf life was limited to 12 months from the time of production [50]. Shott et al. (2012) noted that, quality management systems of POC diagnostics can really transform the availability of tests in real time to inform proper patient care [53]. Jaya et al. (2017) study findings in primary healthcare clinics in rural KwaZulu-Natal in South Africa showed 4 out of eleven clinics reported past experiences of HIV rapid test kits shortage [46]. Three of the 4 clinics, informed patients when there was a shortage and asked them to return at a later date, whilst the fourth clinic referred the patients to neighbouring healthcare facilities [46]. A study aimed to highlight challenges faced in decentralizing POC testing showed that access to HIV testing can be challenging when the service is only available at the national level [56]. It also demonstrated that long turn-around time for release of test results accounted for a significant proportion of people failing to return for test results in LMICs [56]. These findings reveal stock-outs of HIV and HBV test kits, CD4 test, blood pressure monitoring devices, diabetes, and dyslipidaemia POC assays that requires further investigations to ascertain the supply chain management challenges for these diagnostics in LMICs.

##### Reasons for POC diagnostic stock-out

This review revealed that POC test stock-outs mostly was due to poor quantification and forecasting, inventory management of POC diagnostic tests, and inadequate/lack of supervision during implementation [44, 51, 54]; inaccurate documentations and distribution systems [48, 57]; lack of storage space [45]; and poor commodity management [47]. The rest included: high cost and poor quality management systems [52, 53]; poor regulatory controls and limited guideline [52]; and inability of production line to meet global uptake [52]. Therefore, future studies are recommended in LMICs aiming at determining the impact of diagnostic test stock-outs and health outcomes especially for HIV, syphilis, and malaria test.

#### Use of POC diagnostics

Use of POC diagnostics by healthcare workers most often is facilitated by availability of the test ensured through adequate SCM and training. Use of POC diagnostics in this review was reported by 7 out of the 15 studies [43–45, 47–50]. Of the 7 studies, 3 reported malaria RDTs and RST use [43, 49, 51]. The remaining 4 studies reported as follows: limited used of malaria RDTs [45], limited used of blood pressure, diabetes, and dyslipidaemia assays [55], low testing of HBV [44], and low repeated CD4 testing [50].

In a study aimed to assess the quality and safety of having community health workers use rapid diagnostic tests found 96.3% (939/973) of malaria RDTs use for testing children during follow-up visit [43]. Hasselback et al. (2014) found significantly high levels of lost consumption for malaria RDTs ranging from 0 to 149% with an average of 78% weighted by consumption [48]. In addition, malaria RDTs use increased approximately by 300% during rainy seasons [48]. It further reported that consumption was limited up to the point of stock-out [48]. Kyabayinze et al. (2013) found only 20% malaria RDTs use [45]. This outcome demonstrates increased use of malaria RDTs during rainy seasons though usage can also be limited by stock outs, hence needs to be addressed to ensure increased availability of RDTs during rainy season.

##### Use of syphilis, HIV and HBV diagnostics

A research study conducted in Zambia showed increased syphilis testing during the early stages of RSTs introduction but subsequently dropped at the midline and end line [47]. It demonstrated increased syphilis testing from (140/1365, 10.6%) at the baseline to (976/1446, 67.5%) midline, and dropped to (752/1337, 56.3%) at the end line (*P* < 0.001) for both midline and end line compared with the baseline [47]. Mabey et al. (2012) study in Tanzania, Uganda, China, Peru and Zambia and Brazil has shown that more than 100,000 pregnant women were tested for syphilis in all the study site [49]. It also showed that in Brazil, healthcare workers exceeded the original set target (30–40%) in remote communities and succeeded in testing 55% of the sexually active population for syphilis [49]. Smith et al. (2015) indicated that, HIV and syphilis testing uptake by pregnant women was 50.3% HIV and 42.2% for HBV following the introduction of these tests in the antenatal clinic [44]. The study also reports corresponding increases for HIV and syphilis testing uptake as 143.9% (*P* < 0.001) and of 1.3% (*P* = 0.87) respectively [44]. In all, 51.3% (462/901) pregnant women were tested either at the health posts or by outreach teams and 48.7% (439/901) at the district healthcare center [44]. This finding indicates increased use and uptake of syphilis RSTs, HIV and HBV test though use of RSTs declined at some stages. This also demonstrates a gap in literature on the role of SCM for POC diagnostics use which requires further investigations to determine its effect on clients.

##### Use of CD4 diagnostics

Thairu et al. (2011) focused on describing lessons learned in providing CD4 diagnostics in Burkina Faso and Zimbabwe [50]. The study reported that, a total of 3287 CD4 tests were performed for 1558 patients enrolled in the programme in Burkina Faso with an average of two tests per patient (range: 1~10) [50]. In all, 60% of the patients had only one test at the initial stage of the programme but the frequency of repeated tests within 12 months as recommended by national guidelines varied from 59% at the baseline to 16% [50]. A total of 8990 tests were done on 6024 patients in Zimbabwe with an average of one test per patient (range: 1~4) [50]. This outcome demonstrates declined repeated CD4 testing due to poor test stock out.

## Discussion

We conducted a systematic scoping review of studies to explore evidence on supply chain management of and accessibility to POC testing, focusing on availability and use of POC tests in LMICs. The results demonstrated limited published research on SCM of and accessibility to POC testing in LMICs. It also revealed stock-outs of HIV, syphilis, and malaria POC tests. This study findings further demonstrated limited use of malaria RDTs, blood pressure, diabetes, and dyslipidaemia assays, low testing of HBV, and low repeated CD4 testing due to test stock-outs.

Our study findings suggested quantification and forecasting, procurement, inventory management, distribution systems, quality management systems, and human resource capacity played a key role on the availability and use of POC test. Availability of adequate quality POC diagnostic tests essentially increases access to POC testing and improved healthcare. Despite this, evidence from this study showed weak procurement, inventory and stock management, and human resource capacity for SCM resulted in test stock-outs as well as, declined use of RST [47, 51, 54]. The study findings also showed significant stock-outs of malaria RDTs [43, 45, 48], HIV, CD4, HBV POC diagnostics kits [44, 46, 50, 55] at different study settings. This study findings additionally suggested limited use of various POC tests owing to test stock-outs at implementation sites. It is worthwhile to strengthen quantification and forecasting, procurement, inventory management, distribution systems, quality management systems, and human resource capacity to prevent test stock-outs, sustain POC testing services, and maximize the benefits of implementing POC testing programmes in LMICs.

The first WHO model list of essential in-vitro diagnostics (EDL) has been created [7]. The WHO further encourages countries to create national EDLs based on each country’s disease burden and epidemiology [7] to improve access to health care for all patients [44, 49, 51, 52, 56]. Adopting the WHO quality-ASSURED (Affordability, Sensitivity, Specificity, User friendly, Rapid and robust, Equipment free and Delivered) criteria for selection of POC diagnostics for rural and remote settings clinics [58–60] can potentially contribute to addressing SCM challenges to prevent POC test stock-outs and limited usage as evidenced in this study [43–48, 50, 51, 53–55]. All POC testing related services are introduced with the aim of improving health outcomes. For instance, syphilis among pregnant women is highly associated with increased still births [49, 51]. Hence, ensuring availability of syphilis POC tests through efficient SCM during antenatal care potentially could enable syphilis same-day testing and treatment for pregnant women and their partners [44, 49, 51]. Also, efficient SCM will enable availability and increase use of malaria POC tests hence, reduce syndromic management by clinician and prevent wrong treatment or overtreatment of patients related to syndromic management [13, 58, 61]. HIV testing also serves as a gateway to HIV/AIDS prevention, care, and treatment as well as other needed supportive health care interventions [44, 56, 62, 63]. In view of this, decentralizing HIV access to lower health facilities will help improve access to HIV testing and early initiation of management [52, 56]. Unlike, syphilis, malaria, HIV, and other POC diagnostic tests, CD4 count testing is aimed at improving the health outcomes of people living with HIV/AIDs by enabling the determination of clinical staging for the appropriate antiretroviral treatment [64–67]. Therefore, ensuring availability, use, and sustainability of HIV and CD4 testing diagnostic services in LMICs as evidenced in this study is a necessity [50].

### Implications for practice

Majority of the studies reviewed were conducted in a rural setting where access to healthcare and laboratory infrastructure is either not available or poorly developed coupled with lack/inadequate skilled healthcare professional [43–45, 47–51]. This study finding indicates majority of the reviewed studies reported poor availability or stock out of test at the district and primary healthcare settings [43–48, 50–52, 54, 55]. This implies that rural populations including pregnant women and children will have to travel long distances to access diagnostic services. Long distances from rural communities to access laboratory services results in delays, long turnaround time, and failure of people to return for their results [56, 68]. Therefore, sustainability of POC diagnostic services in resource-limited settings and rural health facilities is crucial to improve health outcomes in rural communities [16]. Supply chain management measures such as increase production of POC tests to meet global demand, appropriate quantification and forecasting, strengthening procurement, adequate quality assurance/control, equitable distribution, inventory management; training, among others are highly essential to ensure sustainability of a POC diagnostic service.

### Implications for research

Our study shows that there is limited published research specific to SCM for POC diagnostics in LMICs, indicating a gap in literature. Our study findings also have international implications since the included studies were from diverse different countries. We hope this study will prompt further studies to provide a contextual insight on SCM for POC diagnostics bearing in mind the need to improve access for POC diagnostics services in LMICs. We further recommend a study to determine the impact of POC test stock-outs on patient outcome.

### Strengths and limitations

This scoping review possibly is the first comprehensive study to map evidence on SCM and accessibility to diagnostic services at POC in LMICs. This study demonstrated a substantial gap in literature on POC tests SCM to guide future research in LMICs. This study’s methodology also allowed the inclusion of different study designs and identification of relevant articles methodically, charting, and analysing the outcomes [21, 69]. The removal of date, language, and study design limitations as well as the comprehensive search for literature is an important strength of this study Despite this, it is possible research on POC tests SCM probably existed under different terminologies which were not captured in this review. Nonetheless, we included Medical Subject Heading terms to address this. Further analysis of included quantitative studies using meta-analysis could provide more information. However, the exploratory nature of scoping reviews does not involve meta-analysis for quantitative studies. Future studies could focus on meta-analysis. Although many other factors such as financial constraints, geographical barriers, and human resource capacity for POC testing also play a crucial role regarding access to POC testing [9, 12, 13], this study was limited to only SCM. We therefore recommend similar studies to investigate the role of other essential potential barriers in LMICs in future.

## Conclusion

In conclusion, this study demonstrated limited published research on SCM of and accessibility to POC testing in LMICs. SCM play an essential role on the availability and use of POC tests as well as improved access to healthcare in resource-limited settings. Hence, it is worthwhile to consider SCM as a key component of POC diagnostic service implementation at the planning stages to ensure sustainability. We therefore, recommend primary studies aimed at investigating SCM of POC diagnostic tests in resource-limited settings to identify the barriers/challenges, and provide a context-specific evidence-based solutions for policy/decision makers, implementers, and POC developers, funders, and development partners.

## Additional files

Additional file 1: Electronic databases search results for title screening. (DOCX 14 kb)

Additional file 2: Full articles screening results and output of degree of agreement in Stata Version 13. (DOCX 16 kb)

## Data Availability

The data supporting the conclusions of this paper are available through the detailed reference list. No original datasets are presented, since this is a review of already existing literature.

## Abbreviations

AIDS: Acquired immuno-deficiency syndrome
ASSURED: Affordability, Sensitivity, Specificity, User friendly, Rapid and robust, Equipment free, and Delivered
CD4: T lymphocytes
CHW: Community health workers
EDL: List of essential in-vitro diagnostics
HBV: Hepatitis b virus
HIV: Human immune-deficiency virus
LMICs: Low- and middle-income countries
MMAT: Mixed method appraisal tool
PICO: Population, intervention, comparison, and outcome
POC: Point-of-care
PRISMA: Preferred reporting items for systematic reviews and meta-Analyses
PROSPERO: International prospective register of systematic reviews
RDTs: Rapid diagnostic tests
RSTs: Rapid syphilis tests
SCM: Supply chain management
VL: Viral load
WHO: World Health Organization
