# Supplementary material for: Supply chain management and accessibility to point-of-care testing in resource-limited settings: a systematic scoping review
Source: BMC Health Serv Res. 2019 Jul 24;19:519. doi: 10.1186/s12913-019-4351-3 (PMC6657084; doi:10.1186/s12913-019-4351-3)
Supplement: Supplementary file 1 — Electronic databases search results for title screening. (DOCX 14 kb) [file 12913_2019_4351_MOESM1_ESM.docx]

**Supplementary file 1: Electronic databases search results for title screening**

| **Date** | **Databases** | **Keywords** | **Search results** | **Eligible studies** |
| --- | --- | --- | --- | --- |
| 16/06/2017 | PubMed | Diagnostics, supply chain | 63 | 11 |
| 16/06/2017 | EBSCOhost (CINAHL and MEDLINE with full text) | Diagnostics, supply chain | 43 | 25 |
| 16/062017 | Web of science | POC diagnostics, supply chain management | 1,104 | 40 |
| 16/06/2017 | Science Direct | POC diagnostics, supply chain management | 973 | 51 |
| 17/06/2017 | Google Scholar | POC diagnostics, supply chain management | 19,600 | 180 |
| **TOTAL** |  |  | 21,783 | 307 |
