# Supplementary material for: Supply chain management and accessibility to point-of-care testing in resource-limited settings: a systematic scoping review
Source: BMC Health Serv Res. 2019 Jul 24;19:519. doi: 10.1186/s12913-019-4351-3 (PMC6657084; doi:10.1186/s12913-019-4351-3)
Supplement: Supplementary file 2 — Full articles screening results and output of degree of agreement in Stata Version 13. (DOCX 16 kb) [file 12913_2019_4351_MOESM2_ESM.docx]

**Supplementary file 3: Full articles screening results and output of degree of agreement in Stata Version 13**

| **Author and date** | **First reviewer** | **Second reviewer** |
| --- | --- | --- |
| Albert, 2017 | No | No |
| Alemnji, 2011 | No | Yes |
| Alemnji, 2014 | No | Yes |
| Manso, 2013 | No | No |
| Ansbro. 2015 | Yes | Yes |
| Atun, 2010 | No | No |
| Baltacioglu, 2007 | No | No |
| Bonawitzs, 2015 | Yes | Yes |
| Bristow, 2015 | No | No |
| CHEGE, 2012 | No | No |
| Fu, 2011 | No | No |
| Hamer, 2012 | Yes | Yes |
| Hasselbeck, 2014 | Yes | Yes |
| Jamieson, 2016 | No | No |
| Kyabazinze, 2012 | Yes | Yes |
| Mabey, 2012 | Yes | Yes |
| Maltha, 2013 | No | No |
| McGuire, 2014 | Yes | Yes |
| Park, 2012 | No | No |
| Peeling, 2015 | Yes | Yes |
| Peter, 2009 | No | No |
| Piatek, 2013 | No | No |
| Raja and Bates , 2009 | No | No |
| Shott, 2012 | Yes | Yes |
| Sinishaw, 2015 | No | No |
| Smith, 2015 | Yes | Yes |
| Stevens, 2014 | Yes | Yes |
| Thairu, 2011 | No | Yes |
| Williams, 2016 | No | No |
| Peeling, 2009 | Yes | Yes |

**. kap Firstreviewer Secondreviewer**

Expected

Agreement Agreement Kappa Std. Err. Z Prob>Z

-----------------------------------------------------------------

90.00% 50.00% 0.8000 0.1789 4.47 0.0000

**mcc Firstreviewer Secondreviewer**

| Controls |

Cases | Exposed Unexposed | Total

-----------------+------------------------+------------

Exposed | 12 0 | 12

Unexposed | 3 15 | 18

-----------------+------------------------+------------

Total | 15 15 | 30

McNemar's chi2(1) = 3.00 Prob > chi2 = 0.0833

Exact McNemar significance probability = 0.2500

Proportion with factor

Cases .4

Controls .5 [95% Conf. Interval]

--------- --------------------

difference -.1 -.240685 .040685

ratio .8 .6211555 1.030338

rel. diff. -.2 -.447918 .047918

odds ratio 0 0 2.419952 (exact)
